# Contributions to the Flora of Tropical East Africa

**DOI:** 10.3390/plants12061336

**Published:** 2023-03-16

**Authors:** Shenglan Du, Miaoxuan Wang, Neng Wei, Geoffrey Mwachala, Guangwan Hu, Lin Wu, Shengwei Wang, Qingfeng Wang

**Affiliations:** 1 College of Forestry and Horticulture, Hubei Minzu University, Enshi 445002, China; 2 Key Laboratory of Plant Germplasm Enhancement and Specialty Agriculture, Wuhan Botanical Garden, Chinese Academy of Sciences, Wuhan 430074, China; 3 Sino-Africa Joint Research Center, Chinese Academy of Sciences, Wuhan 430074, China; 4 Center of Conservation Biology, Core Botanical Gardens, Chinese Academy of Sciences, Wuhan 430074, China; 5 East African Herbarium, National Museums of Kenya, Nairobi P.O. Box 45166 00100, Kenya

**Keywords:** conservation, flora, new taxa, plant diversity, tropical East Africa

## Abstract

Tropical East Africa (TEA) is one of the most important biodiversity hotspots on the planet. Its rich flora diversity and inventory have been clearly recognized after the publication of the last volume of the *Flora of Tropical East Africa* (FTEA) in 2012. However, many new and newly recorded taxa have been named and documented since the publication of the first volume of FTEA in 1952. In this study, we comprehensively compiled new taxa and new records by reviewing the literature on the taxonomic contributions of vascular plants in TEA from 1952 to 2022. Our list includes 444 new and newly recorded species belonging to 81 families and 218 genera. Among these taxa, 94.59% of the plants are endemic to TEA and 48.42% are herbs. Additionally, members of Rubiaceae and *Aloe* are the most numerous family and genus respectively. These new taxa are unevenly distributed in TEA, but are found mainly in areas of high species richness, such as coastal, central and western areas of Kenya, central and southeastern Tanzania. This study offers summative assessment of the newly recorded flora inventory in TEA and provides recommendations for future research on plant diversity survey and conservation.

## 1. Introduction

Up-to-date information on biodiversity is essential for the research on macro ecology, evolution and conservation of any region. However, the first step should be to compile a species inventory or checklist [1]. Though many large-scale biodiversity surveys and inventories have been carried out in many regions, new species and records are constantly being reported. Therefore, failure to update biodiversity data promptly will have a negative impact on the accurate understanding of the regional species composition, distribution patterns, and conservation research.

Tropical East Africa (TEA) is one of the most important biodiversity areas in the world, surrounded by three biodiversity hotspots: the Horn of Africa, the Eastern Afromontane, and the Coastal Forests of East Africa [2]. TEA has a high diversity of plants, accounting for about a quarter of tropical plants in Africa [3,4]. In particular, the completion of the *Flora of Tropical East Africa* (FTEA) provides a well-documented checklist of the flora of TEA. FTEA, a project of the Royal Botanic Gardens, Kew, launched in 1948 and completed in 2012 recorded over 12,000 plant species in Uganda, Kenya, and Tanzania [5]. Unquestionably, FTEA is the largest regional tropical flora project ever created. However, it has been more than 40 years since the publication of the first volume (e.g., Oleaceae) [6] in 1952 and 10 years since the publication of the last volume (e.g., Solanaceae) [7] in 2012. In the past 70 years, many new plant species and new records have been discovered and reported in TEA. There are more than 16,100 vascular plants in the TEA, according to our previous research, which were compiled from several online plant databases and the FTEA, belonging to 248 families and 2306 genera [8]. However, we found that not all of the newly published records and new species are currently present in the online databases.

Based on a thorough review of the literature, we have created a checklist of all new and recently discovered taxa that have been documented in TEA from 1952 to 2022 for this study. We anticipate that these floristic and taxonomic contributions will help botanists stay up to date on taxonomic changes in the TEA and provide a solid database for future studies on plant diversity and conservation in the region.

## 2. Results

A total of 444 newly reported species, representing 81 families and 218 genera are presented. 413 new vascular plant species (including 33 subspecies, 19 varieties) and 31 new records (including 2 subspecies, 1 variety) have been documented in TEA. There are 425 angiosperms, 11 pteridophytes and 8 gymnosperms among these newly and recently discovered taxa (Table S1). There are 204 species that have been assigned in the IUCN red list category, of which 41 are critically endangered (CR), 78 endangered (EN), 40 vulnerable (VU), 2 near threatened (NT), 24 least concern (LC) and 19 data deficient (DD) (Table S1).

Some of the newly described genera include: *Hoffmannanthus* H. Rob., S.C. Keeley & Skvarla [9], *Jeffreycia* H. Rob., S.C. Keeley & Skvarla [9], *Mwasumbia* Couvreur & D. M. Johnson [10], *Kylicanthe* Descourvières, Stévart & Droissart [11], *Lukea* Gosline & Cheek [12], *Normantha* P.J.D.Winter & B-E.van Wyk [13], *Kenyacanthus* I. Darbysh. & Kiel [14] and *Zulustylis* Muasya [15]. The seven newly recorded genera are *Calyptocarpus* Less. [16], *Argyrella* Naudin, Gastrodia R.Br. [17], *Hymenasplenium* Hayata [18], *Hypseochloa* C.E.Hubb. [19], *Mischogyne* Exell [20] and *Moeroris* Raf. [21] (Table 1).

In this checklist, the top ten families with the highest species richness include: Rubiaceae (40 species (spp.) in 15 genera (gen.)), Fabaceae (36 spp. in 21 gen.), Annonaceae (35 spp. in 15 gen.), Asphodelaceae (30 spp. in 1 gen.), Euphorbiaceae (30 spp. in 10 gen.), Asparagaceae (26 spp. in 3 gen.), Orchidaceae (26 spp. in 17 gen.), Cucurbitaceae (19 spp. in 7 gen.), Poaceae (19 spp. in 14 gen.), and Phyllanthaceae (9 spp. in 3 gen.). The top ten genera with the highest species richness are *Aloe* (Asphodelaceae/30 spp.), *Dracaena* (Asparagaceae/21 spp.), *Euphorbia* (Euphorbiaceae/18 spp.), *Pavetta* (Rubiaceae/10 spp.), *Encephalartos* (Zamiaceae/8 spp.), *Lobelia* (Campanulaceae/8 spp.), *Coffea* (Rubiaceae/7 spp.), *Crotalaria* (Fabaceae/7 spp.), *Impatiens* (Balsaminaceae/7 spp.), *Asplenium* (Aspleniaceae/6 spp.), *Indigofera* (Fabaceae/6 spp.), *Phyllanthus* (Phyllanthaceae/6 spp.), *Polystachya* (Orchidaceae/6 spp.), *Xylopia* (Annonaceae/6 spp.) and *Zehneria* (Cucurbitaceae/6 spp.) (Table S1).

**Figure 1:**
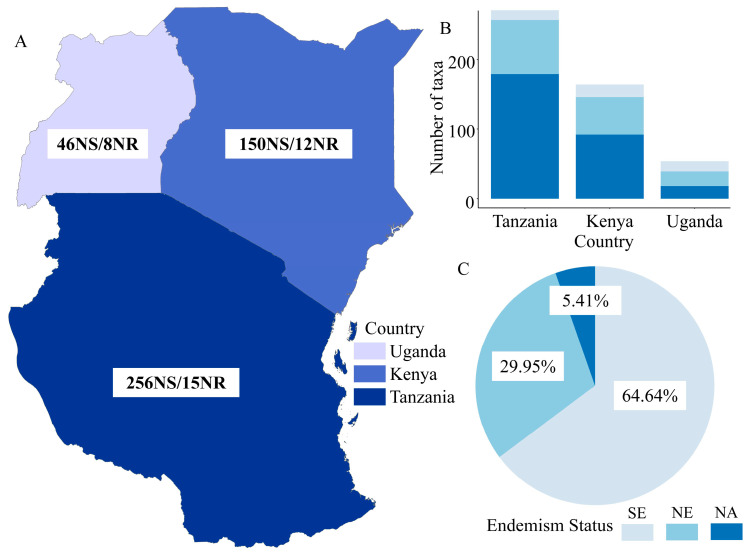
Overview of new and newly recorded taxa and endemism of plants in tropical East Africa countries. (**A**) Number of new and newly recorded taxa in each country; (**B**) Number of new endemic taxa in each country; (**C**) Proportions of different endemism in TEA. NS: new species; NR: new records; SE: strictly endemic; NE: near-endemic; NA: non-endemic.

Tanzania has the highest number of new taxonomic contributions with a total of 271 taxa (256 new species (NS) and 15 new records (NR)), followed by Kenya 162 taxa (150 NS/12 NR), Uganda 54 taxa (46 NS/8 NR) (Figure 1A). Among them, Tanzania has the highest number of new endemic species (179 SE/78 NE), followed by Kenya (90 SE/54 NE), and Uganda (18 SE/21 NE) (Figure 1B). In general, the endemism of new taxa in TEA is high, about 94.59%, of which 64.64% are SE and 29.95% are NE (Figure 1C).

**Figure 2:**
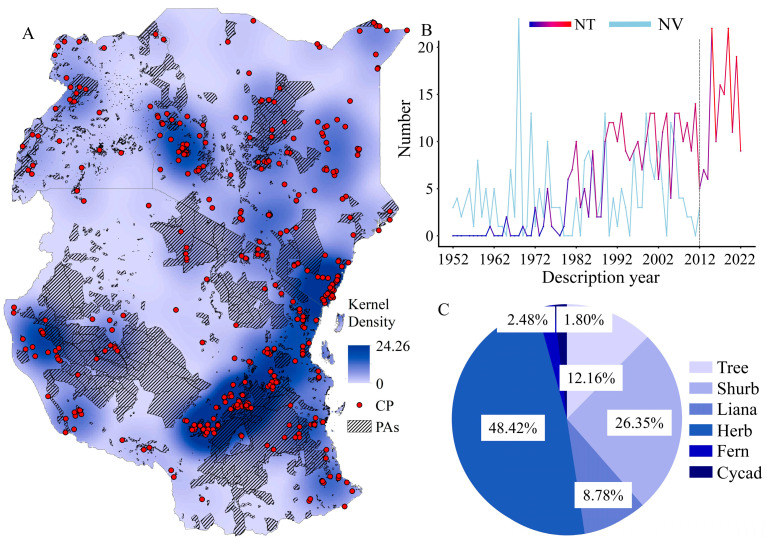
(**A**) Distribution pattern and kernel density of new and newly recorded taxa in tropical East Africa; (**B**) Number of new publication. The blue to red line shows the number of new and newly recorded taxa over the past 61 years (1961–2022) in tropical East Africa, the light blue line shows the number of published volume for the *Flora of Tropical East Africa* from 1952 to 2012; (**C**) Proportion of the growth habit and life form for the new and newly recorded taxa in tropical East Africa (Protected area data from the World’s protected areas (https://www.protectedplanet.net/en, accessed on 2 November 2022). The dotted line shows the year 2012 when the last volume of the *Flora of Tropical East Africa* was published. PAs: protected areas; CP: collection point; NT: number of new taxa; NV: number of published volume.

Over the past 70 years, the spatial and temporal distribution of new and newly recorded taxa in TEA has been uneven. Kernel density analysis shows that high-density areas are mainly in coastal, central and western areas of Kenya; central and southeastern Tanzania (Figure 2A). Several new and newly recorded taxa were documented between 1952 and 1961, though most of them had been documented in the FTEA. Therefore, in this study, the earliest new taxon was recorded in 1961 [22]. The number of new and newly recorded taxa increased irregularly from 1961. However, since 1989, more than 5 new and newly recorded taxa have been reported almost every year (except for 2005 and 2012), and the highest number of new taxa was reported in 2015 and 2019 (22 spp.) (Figure 2B).

The growth habit and life forms of the new and newly recorded taxa in TEA are diverse but the proportion is uneven among them, herbs accounted for the highest number (48.42%), followed by shrubs (26.35%), trees (12.16%), lianas (8.78%), ferns (2.48%), and cycads (1.80%) (Figure 2C).

## 3. Discussion

The updating of new species and newly recorded taxa can help clarify the richness and inventory of the local flora, and can also offer suggestions for future taxonomic research [23,24,25]. In this study, 444 new and newly recorded species in TEA from 1952 to 2022 are documented. The identification of these new species is largely a result of the ongoing taxonomic revision and the supportive contribution of recent molecular studies, which have updated the geographical data of some genera and species as well as the status of previously published species. These plant lists will be extremely helpful in understanding the evolution of taxonomic studies on the flora of TEA.

In this study, the number of new taxa and new records is relatively low compared to the number of new taxa discovered in Africa (According to IPNI [26], more than 2900 new taxa have been discovered since 2021 and 5300 new taxa have been documented since 2000 in Africa). The low number counted in this study may also be related to the shorter period for FTEA to be completed, and the sharp increase in the number also occurred after the completion of FTEA (2012) (Figure 2B), which verifies that the FTEA team conducted a very extensive and comprehensive flora inventory. However, a portion of unidentified species are also recorded in FTEA, and these species are documented as *sp A*, *sp B*..., and their number may exceed 500 species. Moreover, about 16% area of TEA has no records of plant specimen collection, and more than 50% area is described as an incomplete collection [8]. As a result, we think that TEA still has a lot of novel taxa waiting to be found. Empirically and theoretically, families with more species have a higher probability of containing undescribed species. However, Orchidaceae and Fabaceae are exceptional, the number of megadiverse families such as Poaceae and Asteraceae did not show this regularity. The same conclusion appeared in the statistics of new taxa in Myanmar [27]. This may be attributed to the discoverer’s research interest. Undeniably, those highly ornamental flora (e.g., Orchidaceae, *Impatiens*) and succulents (e.g., *Aloe*, *Dracaena*, *Euphorbia*) have gained more attention from researchers. Additionally, this may also be caused by the differences in the taxonomic expertise of the discoverers, because the more species the family has, the more difficult it is to identify, such as Poaceae and Cyperaceae. What’s more, it is difficult for a country to have experts on all taxa, so extensive collaborations and exchanges among international taxonomists are indispensable [27,28]. Furthermore, with the widespread use of molecular biology in taxonomy, it will effectively help taxonomists to find potential new species in those families and genera that are difficult to identify.

Herbaceous plants are the most abundant flora life forms among new and newly recorded taxa discovered in TEA, and mainly distributed in Orchidaceae, Asphodelaceae, Asparagaceae, and Cyperaceae. Compared to tall trees, herbaceous plants are easier to be collected as specimens and more convenient for ex situ cultivation for further observation. Meanwhile, since most of the TEA region is in an arid climate zone [29], many species have underground succulent rhizomes to adapt to the prolonged dry season. These plants can only be found when they bloom in or after the rainy season, such as *Eriospermum adpressifolium* O. Weber (Asparagaceae) [30], *Emilia blittersdorffii* Beentje (Asteraceae) [30], and *Zygotritonia teretifolia* Goldblatt & J.C. Manning (Iridaceae) [31]. The new woody plants obtained in this study are mainly concentrated in the Rubiaceae (40 spp.) and Annonaceae (35 spp.), which may be due to the fact that these two families are widely distributed in tropical and subtropical regions of the world and they all hold a large number of species.

Areas with higher densities of new and newly recorded taxa in TEA coincide with the areas with high species richness, such as coastal forests, western Kenya, southeastern and central-western Tanzania (Figure 2A). Collectors are more willing to conduct surveys and specimen collection in areas with high species richness or convenient transportation [32,33,34], which increases the likelihood of discovering new species and suggests that areas with high species richness and where multiple surveys have been conducted still need to be surveyed [8]. However, many new species have also been discovered in savannah with low plant diversity, and almost all of them are strictly endemic, such as *Sansevieria marachiensis* T.G.Forrest (Asparagaceae) [35], *Aloe lukeana* T.C. Cole (Asphodelaceae) [36], *Kyllinga mbitheana* Muasya (Cyperaceae) [37], and *Cyperus volkielloides* Muasya & Vollesen (Cyperaceae) [38]. There are many areas in the savannah of TEA that have never been traced by flora collectors [8], and a comprehensive flora inventory is seriously lacking. Researchers are always fascinated by diversity hotspots areas, but they should not overlook the plant diversity in vast savannah, and there are certainly novel species awaiting to be discovered by taxonomists.

The acceleration of economic integration, the growing process of international trade liberalization and the intensification of human activities has created conditions for the long-distance migration and invasion, spread and dispersal of exotic species [39]. The risk of invasion of exotic organisms is increasing [40]. The invasive species are causing serious harm to local society, economy and even human health by changing the natural ecosystem of the invasion site and reducing species diversity [41,42]. Therefore, more attention should be paid to those exotic species that are not yet defined as invasive. Meanwhile invasion assessment and prevention measures should be carried out for exotic plants as soon as possible. Moreover, the new and existing exotic plants in TEA must be highly valued, especially the assessment of their invasion and potential impact on native plants.

More than half of the new and newly recorded plant taxa in TEA have not been assessed by the IUCN. Even among the 204 plants that have been assigned an IUCN category, there are still 48 species for which only the recommended IUCN category has been recorded in the literature. Incomplete information on species distribution as well as population size is an important factor affecting the assessment of species endangerment levels [43,44]. Many taxonomists tend to focus more on the scientific aspects of taxonomy when conducting plant diversity surveys, and neglect the collection of information on species population groups. However, it is undeniable that it is the small population size, narrow distribution area and special habitats of many plants that make them less easily found [43]. These plants are often more vulnerable to environmental changes and more highly endangered [45]. Therefore, the investigation of population and distribution data of these new taxa not assessed by IUCN is urgent, which can enable them to be seriously and effectively protected as soon as possible. The investigation of population and distribution data of these new taxa is urgently needed. Early assessment of these new taxa according to IUCN criteria can enable them to be conserved carefully and effectively. At the same time, for those new plant taxa that have been listed in the IUCN category, we should also promptly promote research on their endangerment mechanisms and effective conservation.

**Figure 3:**
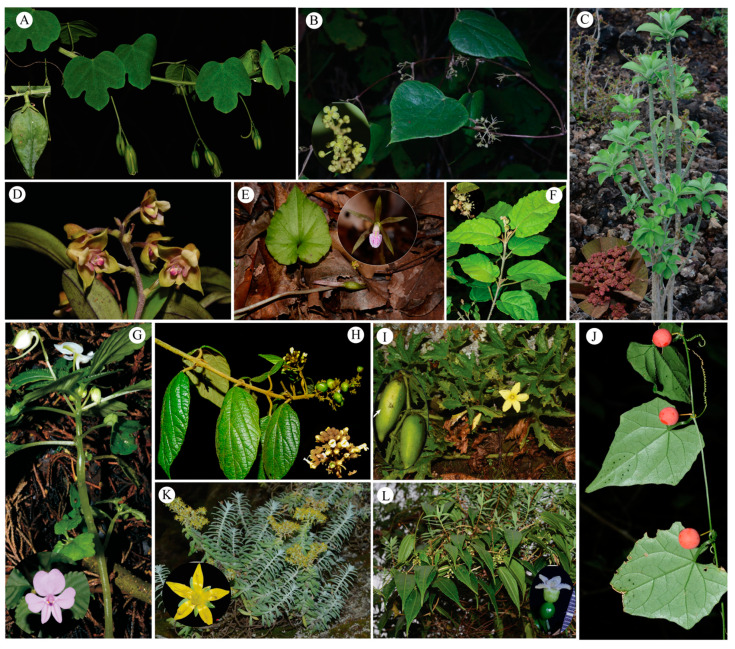
Some noteworthy new and recently discovered taxa found in tropical East Africa by SAJOREC team. (**A**) *Adenia angulosa* G.W. Hu & Q.F. Wang, 2017; (**B**) *Cissampelos keniensis* Y.D. Zhou & Q.F. Wang, 2017; (**C**) *Euphorbia mbuinzauensis* N. Wei, Mwachala, G.W. Hu & Q.F. Wang, 2021; (**D**) *Polystachya danielana* G.W. Hu, W.C. Huang & Q.F. Wang, 2019; (**E**) *Nervilia lilacea* Jum. & H. Perrier, 2019; (**F**) *Croton kinondoensis* G.W. Hu, Ngumbau & Q.F. Wang, 2020; (**G**) *Impatiens pseudoviola* Gilg var. alba G.W.Hu & Q.F. Wang, 2017; (**H**) *Premna mwadimei* V.M. Ngumbau & G.W. Hu, 2021; (**I**) *Peponium elgonense* Neng Wei, G.W.Hu & Q.F. Wang, 2020; (**J**) *Zehneria monocarpa* G.W. Hu, V.M. Ngumbau & Q.F. Wang, 2020; (**K**) *Sedum keniense* Y.D. Zhou, G.W. Hu & Q.F. Wang, 2016; (**L**) *Zehneria subcoriacea* Y.D. Zhou & Q.F. Wang, 2016.

Taxonomists have the chance to discover new species, yet many species may vanish before that window of time. Therefore, the documentation of new species is of great importance for the conservation of species [46]. Many of the new species in TEA are strictly endemic and distributed in a very limited area, such as *Euphorbia mbuinzauensis* N. Wei, Mwachala, G.W. Hu & Q.F. Wang (Euphorbiaceae) [47], which was only found in woodlands covered by lava outcrops with one population. Other newly discovered species are restricted in highly threatened woodland and unprotected areas, for example, *Aloe ngutwaensis* Mwadime & Matheka [48], *Dorstenia arachniformis* Matheka, Malombe, Mwadime & Mwachala [49] and *Premna mwadimei* V.M. Ngumbau & G.W. Hu [50]. Actually, *Premna mwadimei* is only found in one population with only three individuals around farmland [50]. This are just but few example from Kenya. These narrowly distributed taxa are at highest risk of extinction by habitat destruction or loss from grazing, charcoal burning, logging, mining, and climate change [51,52,53,54]. Moreover, as it is shown in our results, many new taxa, especially endemic species, are not in the current protected areas (Figure 2A). Because of the incompleteness of the species inventory survey, the accuracy of the assessment of the survival status and endangerment of the new taxa also needs to be improved. Thus, extensive plant diversity surveys and substantial international collaboration can provide opportunities for the conservation of plant diversity in TEA. For example, the establishment of the Sino-Africa Joint Research Centre of the Chinese Academy of Sciences (SAJOREC) in 2013, and the launch of the project of the Flora of Kenya in 2015, a collaboration between Chinese and Kenyan botanists. So far, the SAJOREC Research Team has conducted floristic studies in numerous plant diversity hotspots [55,56,57,58,59] and discovered 18 new and newly recorded taxa in TEA (Figure 3). Such collaboration will not only promote the understanding and conservation of plant diversity in TEA, but also promote the training of local young taxonomists.

Inventory renewal and conservation of plant diversity in TEA face challenges and opportunities. Here, we call for: (1) strengthening the collaboration of taxonomists worldwide; (2) training more local taxonomists, especially young researchers; (3) conducting more flora inventory surveys; (4) strengthening the plant diversity survey in savannah; (5) accurately assessing populations, habitats, and endangerment of discovered new taxa; (6) strengthening research on in situ and ex situ conservation of new taxa; and (7) strengthening the assessment and expansion studies of current protected areas, etc., to make more contributions to the flora of TEA.

## 4. Materials and Methods

### 4.1. Study Area

**Figure 4:**
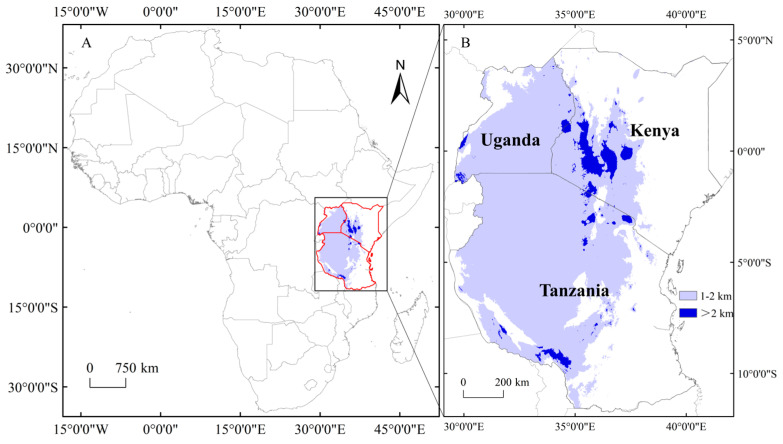
Map of tropical East Africa. (**A**) Geographical location of tropical East Africa; (**B**) National composition. The light blue and blue areas on the map indicate high-altitude areas.

According to the FTEA, Tropical East Africa (TEA) is defined as the regions of Kenya, Uganda and Tanzania. This region occupies an area of 1,768,706 km^2^ and extends from 5°2′ N to 11°45′ S and 29°20′ E to 41°54′ E (Figure 4). Mountains with an altitude of more than 2000 m are mainly concentrated in western Kenya, northern and southwestern Tanzania. TEA has a high plant diversity, accounting for about a quarter of all tropical African plant species, and the hotspots of plant biodiversity are mainly distributed in these alpine groups and coastal forests in East Africa [8].

### 4.2. Data Collection

In this study, we defined a new taxon as a species that was not documented referring to the publication of the section which belongs to FTEA. If it was the first time for a species to be documented in TEA, we defined it as a new species (NS). If the species had new distribution record in TEA, we defined it as new record (NR). The taxonomic data for NS and NR in TEA were assembled from published taxonomic literature from January 1952 to December 2022. Keywords such as (new species, new record, new genus, new family, new taxon and Kenya or Uganda, Tanzania, East Africa and tropical East Africa) were searched on major academic online resources, such as Google and Google Scholar, Web of Science, Research Gate, and others. Then, we looked for plants for each country using the International Plant Names Index (IPNI, https://www.ipni.org/, accessed on 15 March 2022) [26] and compared plant species with the Plants of the World Online (POWO; https://powo.science.kew.org/, accessed on 15 March 2022). Finally, the species that were not listed in the FTEA were noted in our checklists (replicated species were kept only once).

### 4.3. Checklist Outline

The correct taxonomic names (families and genera) of the obtained online checklists was derived from the “plantR” R package [60], which followed APG IV for angiosperms [61] and PPG I for ferns and lycophytes [62], and Gymnosperm according to Christenhusz et al. [63]. We checked the information of each taxon in the checklist and recorded its distribution information, growth habit, life form, endemism and IUCN red list category. The growth pattern and life form of each taxon in this study were recorded by a simple classification, with six main categories: tree, shrub, liana, herb, fern, and cycad [64]. The endemism of taxa was determined with reference to the criteria proposed by Darbyshire et al. [64]. A species was considered to be strictly endemic (SE) if it exclusively occurred in only one national region. Those species that occurred in two or more African countries but not more than five were defined as near-endemic (NE) and others were non-endemic (NA) species. The IUCN category of each taxon was derived from IUCN red list (https://www.iucnredlist.org/, accessed on 12 March 2023) and literature, with nine categories: data deficient (DD), least concern (LC), near threatened (NT), vulnerable (VU), endangered (EN), critically endangered (CR), extinct in the wild (EW), extinct (EX) and not evaluated (NE).

Kernel density analysis and figures related to geographic maps were plotted in ArcGIS 10.2 and statistical graphs were drawn in R 4.0.5 [65].

## Figures and Tables

**Table 1:** Overview data for newly discovered and new records of vascular plants genera in tropical East Africa from 1952 to 2022.

Family	Genus	Category
Acanthaceae	*Kenyacanthus* I.Darbysh. & Kiel	New genus
Annonaceae	*Mwasumbia* Couvreur & D. M. Johnson	New genus
Annonaceae	*Lukea* Gosline & Cheek	New genus
Annonaceae	*Mischogyne* Exell	New recorded genus
Apiaceae	*Normantha* P.J.D.Winter & B-E.van Wyk	New genus
Aspleniaceae	*Hymenasplenium* Hayata	New recorded genus
Asteraceae	*Calyptocarpus* Less.	New recorded genus
Asteraceae	*Hoffmannanthus* H. Rob., S.C. Keeley & Skvarla	New genus
Asteraceae	*Jeffreycia* H. Rob., S.C. Keeley & Skvarla	New genus
Cyperaceae	*Zulustylis* Muasya	New genus
Fabaceae	*Hypseochloa* C.E.Hubb.	New recorded genus
Melastomataceae	*Argyrella* Naudin	New recorded genus
Orchidaceae	*Kylicanthe* Descourvières, Stévart & Droissart	New genus
Orchidaceae	*Gastrodia* R.Br.	New recorded genus
Phyllanthaceae	*Moeroris* Raf.	New recorded genus

## Data Availability

Not applicable.

## Supplementary Materials

The following supporting information can be downloaded at: https://www.mdpi.com/article/10.3390/plants12061336/s1, Table S1. “New and newly recorded taxa in tropical East Africa” and “New and newly recorded genera in tropical East Africa”.
